# Fruit as a Food

**Published:** 1889-10

**Authors:** 


### FRUIT AS A FOOD.

What shall we eat? This question confronts us daily. Upon its wise solution depend to a great extent, the health and happiness of the human race. A judicious dietary is an evidence of a high state of civilization, for brain and brawn are in a general sense the direct outcome of the kind of food eaten, its method of preparation, and the style in which it is served and introduced into the human economy. Americans are a little astray in the matter of diet. Having no national type of anything it is not surprising that the characteristic food of the nation is conspicuous by its absence. The average table is a strage mixture of English, Dutch, French and everything else beside, according to whatever polyglot ancestry the individual householder may posess. Even "Ah Sin" has his finger in the pie. The English imprint, however, is the strongest; and, like the English, we have a diet adapted in general to a far colder latitude than the one we inhabit. There seems to be a strong tendency in human nature towards the consumption of food that is too concentrated. The old Indian chief complained that the pale faces (especlally woman) were dying of too much house. The modern civilized world is dying of too much meat.

Some people are afraid to eat fruit, thinking that fruit and diarrhœa are always associated, when if they understood the true cause of the diarrhœa they would know that is was caused by eating meat. In hot weather meat putrifies very quickly, and during this process alkaloids are formed which are very poisonous, acting as emetics and purgatives.

T'is true that fruit eaten green or between meals will interfere with digestion and cause bowel troubles ; but use fruit that is perfectly ripe at mealtime, and only beneficial results will follow. Acids prevent calcareous degenerations, keeping the bones elastic, as well as preventing the accumulation of earthy matters. This is because of the solvent power of the acids ; but manufactured acids are not harmless, as are those which nature has prepared for us in the various kinds of fruit. Fruit is perfect food when fully ripe, but if it were in daily use from youth to age there would be less gout, gallstones and stone in the bladder

Stewed apples, pears and plums are favorite articles of diet. For breakfast or luncheon, in the dining room or in the nursery, there are few table dishes more wholesome and more delicious than well-stewed fruit served up with cream or custard. There are many persons, however, who cannot eat it on account either of the acidity of the fruit or the excess of sugar necessary to make it palatable. Sugar does not, of course, counteract acidity ; it only disguises it, and its use in large quantities is calculated to retard digestion. The house-wife may, therefore, be grateful for the reminder that a pinch—a very small pinch —of carbonate of soda, sprinkled over the fruit previously to cooking will save sugar, and will render the dish at once more palatable and more wholesome.

Taken in the morning, fruit is as helpful to digestion as it is refreshing. The newly awakened function finds it an object of such light labor as will exercise without seriously taxing its energies, and the tissues of the stomach acquire at little cost a gain of nourishment which will sustain those energies in later and more serious operations. It is an excellent plan, with this object in view, to add a little bread to the fruit eaten. While admitting its posession of these valuable qualities, however, and while also agreeing with those who maintain that in summsr meat should be less, and fruit and vegetables more freely used as a food, we are not prepared to allow that even than an exclusively vegetarian regimen is that most generally advsiable. Meat provides us with a means of obtaining albuminoid material, which is indispensable in its most easily assimilable form. It affords us in this material not only an important constituent of tissues growth, but a potent excitant of the whole process of nutrition. It has a definite and important place in the ordinary diet of man, and the wholesomeness of fruit combined with farinaceous food as an alternative dietary is not so much an argument in favor of the vegetarian principle, as proof that seasonable changes in food supply are helpful to the digestive processes and to nutritive changes in the tissues generally.

With proper eating and drinking, there would be fewer broken down nervous wrecks, and far more vigorous intellects. The present human species cannot eliminate flesh entirely from its food and amount to a row of pins. The fancy that nothing but vegetables should be eaten is apt to overtake every one somewhere in life. It is due to some physical disorganization, to some passing "kink" or notion, and usually steals silently away with the disturbance that created it. Still, there is far too much meat eaten. Meat three times a day is more than average town-dwelling human nature can endure. Functional disturbances of of the liver, gall-stones, renal calculi, diseases of the kidney, dyspepsia, headache, fits of ill-temper or of the blues, irritability and general absence of the joy of life are largely due to an excess of meat and other highly concentrated food. These conditions, like attacks of gout and dyspepsia, are the unfortunate posession of those who will have them. Ignorance of the law is no excuse. What shall wo eat? As a preliminary answer we reply—eat more fruit.—*Medical Classics.*

